# Acute health service use among people who died from methamphetamine-related causes in New South Wales and Victoria

**DOI:** 10.1186/s12913-026-14952-x

**Published:** 2026-06-18

**Authors:** Oisin Stronach, Paul Dietze, Michael Livingston, Anna Lee Wilkinson, Lauren Moran, Emily Nehme, Amanda Roxburgh

**Affiliations:** 1 School of Public Health and Preventive Medicine, Faculty of Medicine, Nursing and Health Sciences, Monash University, Melbourne, VIC Australia; 2 Disease Elimination, Burnet Institute, Melbourne, VIC Australia; 3 National Drug Research Institute, Curtin University, Perth, WA Australia; 4 Melbourne School of Population and Global Health, University of Melbourne, Melbourne, VIC Australia; 5 Vital Statistics, Australian Bureau of Statistics, Brisbane, Australia; 6 Ambulance Victoria, Melbourne, Australia

**Keywords:** Methamphetamine, Amphetamine, Mortality, Health service use, Epidemiology, Overdose

## Abstract

**Background:**

Despite increased methamphetamine-related harms in Australia, little is known about healthcare contacts amongst those who die from methamphetamine-related causes. The study aims to describe the proportion and incidence rates of acute health service contacts preceding methamphetamine-related death in New South Wales and Victoria.

**Methods:**

We conducted a retrospective analysis of acute health service use among people who died from methamphetamine-related causes in New South Wales and Victoria (2008–2021), using linked ambulance, emergency department, and hospital data (2007–2021). Proportions of individuals with any service contact and incidence rates (contacts per person-year) were calculated and stratified by sex, age and state, across the entire period, 12 months, and 30 days prior to death.

**Results:**

There were 2,075 methamphetamine-related deaths in New South Wales and 2,045 in Victoria between 1 July 2008 and 31 December 2021. Over 90% had at least one acute healthcare contact prior to death, with the emergency department the most common point of contact. Two-thirds had acute service use in the 12 months, and one-third in the 30 days before death. Incidence rates of contacts for each service increased with proximity to death, peaking in the 30 days prior. Increases were greatest for emergency department presentations, ranging from 1.2 to 1.4 contacts per person-year for the entire period before death to 2.4–2.7 in the year before death. Incidence rates of ambulance, emergency department and hospital contacts increased over time in New South Wales and Victoria. A small proportion (8–9%) of decedents had no recorded acute service use.

**Conclusions:**

The high proportion and increasing incidence rates of acute healthcare contact in the year preceding death involving methamphetamines highlights points of engagement where earlier intervention strategies could be strengthened, particularly in emergency departments. Findings suggest the need for coordinated pathways from acute settings into ongoing care for people who use methamphetamine.

**Supplementary Information:**

The online version contains supplementary material available at https://doi.org/10.1186/s12913-026-14952-x.

## Background

Methamphetamine use and related harms are increasing globally [1, 2]. In Australia, estimates from the 2022/23 National Drug Strategy Household Survey suggest that 1.6 million Australians have used methamphetamines in their lifetime, with 200,000 people using them in the past year [3]. Methamphetamine use is associated with a range of acute and chronic harms, including physical health harms (e.g., seizures, strokes, self-harm, blood-borne viruses and cardiovascular disease), mental health conditions (e.g., methamphetamine-induced psychosis, depression and anxiety) that result in or contribute to death [4–7]. Methamphetamine-related mortality in Australia has increased four-fold over the last two decades, largely driven by drug toxicity and suicide, with mortality risk highest among people born between 1962 and 1982 (i.e. Generation X) [8, 9]. 

Methamphetamine use places a large burden on acute health services, including emergency departments (EDs), hospital inpatient services, and ambulance services [10]. Hospital presentations in Australia related to methamphetamine use have steadily increased since the early 2000s, and in 2022 accounted for the largest proportion of drug-related hospitalisations [11, 12]. People presenting to EDs or hospitals for methamphetamine-related issues are more likely to arrive by ambulance and with police involvement compared to those presenting for other drug-related concerns [13]. More frequent methamphetamine use is also associated with higher odds of using high-cost acute care services (e.g. EDs, psychiatric hospital admissions), and lower odds of accessing non-acute services like general practitioners or psychologists compared with people who used methamphetamine less frequently [10]. 

Understanding patterns of acute healthcare contact prior to death is crucial for identifying missed opportunities for engagement and support. A recent Canadian cohort study in British Columbia found that 61% of people who died from stimulant toxicity (including cocaine, methamphetamine, gamma-hydroxybutyrate (GHB), and 3,4-methylenedioxymethamphetamine (MDMA)) had at least one healthcare contact in the year before death, and 27% at least one in the 30 days before death [14]. A similar study of stimulant-related toxicity deaths in Ontario, Canada, found that 20% of people experienced at least one hospital admission for substance-related toxicity in the year prior to death [15]. In the US, 85% of veterans who died from stimulant-related toxicity between 2012 and 2018 had visited a primary care service in the past year. Those who died from combined stimulant and opioid overdoses had even higher service use across all healthcare settings [16]. A Taiwanese study of people with methamphetamine use disorder who died by suicide found that 87% of individuals had utilised a health service in the three months before suicide, with greater use of psychiatric services and EDs compared to people who were alive [17]. These findings highlight the potential for acute healthcare settings to serve as key points of intervention for people at elevated risk of methamphetamine-related mortality.

Existing international research on health service utilisation prior to death has focused on all stimulant-related deaths (including cocaine and MDMA) [14, 15] or has examined healthcare contacts related to particular causes of death, such as drug toxicity [16] or suicide [17]. These studies suggest that many individuals who die from stimulant-related causes have significant contact with healthcare services before their death. However, due to variations in drug markets, healthcare access, and service delivery models across countries there is a need for Australian specific analyses to inform local harm reduction and treatment strategies. This is particularly important in Australia, where crystal methamphetamine is the predominant form of methamphetamine used, and is contributing to rising harms and growing pressure on acute health services [18]. Despite this, there has been no Australian research to date that examines acute healthcare contact prior to death among people who have died from methamphetamine-related causes, regardless of the underlying cause of death. Additionally, no international or Australian studies have stratified methamphetamine-related health service utilisation prior to death by sex, leaving a significant gap in understanding gender-specific service needs and intervention points.

### Aims

Using linked data on people who died from methamphetamine-related causes between 1 July 2008 and 31 December 2021 in New South Wales (NSW) and Victoria (VIC), this study aims to:

1. Determine the characteristics of methamphetamine-related deaths;
2. Estimate the proportion and incidence rates of acute healthcare contact across the entire period, twelve months, and thirty days prior to death, and;
3. Examine trends in the incidence rates of acute healthcare contact prior to death.

## Method

### Study design and setting

This study is a retrospective cohort of people aged 15 years and older who died from methamphetamine-related causes in NSW (Australia’s most populous state) or VIC (Australia’s second most populous state), between 1 July 2008 and 31 December 2021. Methamphetamine-related deaths were defined as those where methamphetamine either directly caused death (such as a drug toxicity) or where it was certified as a contributing factor to death. These deaths were identified by the presence of International Classification of Disease, Tenth Revision (ICD-10) codes T43.6 or F15.0-F15.9 (Supplementary Table 1). The death records of the cohort were linked to a range of administrative health data, including hospital, ED, and ambulance records. The primary outcome was hospital, ED, and ambulance contact before meth/amphetamine-related death for the period 1 July 2007 to 31 December 2021.

### Participants

The cohort includes deaths involving all amphetamine-type stimulants, including amphetamine, methamphetamine, MDMA, fenethylline, ephedrine, and prescribed medications containing methylphenidate (e.g., Ritalin). Methamphetamine is the predominant form of amphetamine used in Australia, and for the purposes of this study, these deaths will be referred to as meth/amphetamine-related deaths [8, 19–21]. Deaths were categorised according to the underlying cause of death (Supplementary Table 2).

### Data linkage

Record linkage was conducted by the specialist jurisdictional linkage agencies in NSW, the Centre for Health Record Linkage (CHeReL) and Victoria, the Centre for Victorian Data Linkage (CVDL) (see Supplementary Fig. 1 for project linked data flow) CHeReL conducts linkage to inpatient hospital, ED and ambulance records held internally within NSW Health using a Master Linkage Key spine, which is constructed used deterministic and probabilistic record linkage methods with identifiable information such as name, address, date of birth and sex (see Supplementary Table 1 for ICD-10 codes). Enduring links are stored within the Master Linkage Key in perpetuity, improving linkage quality for datasets with limited identifiers by drawing on pre-linked arrays (see Irvine and colleagues for full description of CHeReL linkage methods) [22]. CHeReL estimates the false positive rate to be extremely low (0.5%).

The Centre for Victorian Data Linkage (CVDL) provided all VIC deaths among those aged 15 years and over (see Supplementary Fig. 1), and the VIC cohort was selected using the same ICD codes provided to CheReL (see Supplementary Table 1). CVDL linked the cohort to inpatient hospital and ED records using the Victorian Linkage Map [23]. CVDL links records using a combination of deterministic criteria, where selected identifiers (name, address, date of birth and sex) match exactly, and probabilistic criteria, where links are formed when match scores based on agreement and disagreement across identifiers exceed a defined threshold. CVDL undertakes post-linkage quality analyses, including assessment of overlap between datasets, manual sampling of linked records to assess false-positive links against an approximate 2% expected error rate, investigation of unmatched records for potential bias or systematic error, and checks of familial structures where relevant (see Supplementary Table 3 for full details of included data sources) [23]. High-likelihood false-positive linkage errors, which represented 0.9% of records, were removed from the Victorian Linkage Map extracts.

The Victorian cohort was provided by CVDL to Ambulance Victoria, the statewide ambulance service in Victoria, for probabilistic linkage to Victorian ambulance records. Initial deterministic linkage using patient name and date of birth was undertaken to identify exact matches. Subsequently, probabilistic linkage methods were applied to identify additional matches, allowing for fuzzy matching on these variables. Records with a match probability between 80% and 85% underwent manual review to confirm accuracy. Matches with a probability greater than 85% were classified as true matches, whereas those with a probability below 80% were considered non-matches.

### Statistical analysis

Time-at-risk commenced on 1 July 2007 and continued until the date of death. Individuals included in the study died on or after 1 July 2008 and contributed at least one year of follow-up. The date for each acute healthcare contact (ambulance attendances, ED presentations, and hospital admissions) was extracted from linked administrative databases. Acute healthcare contacts were excluded if the individual was already deceased or died during that episode of care. Where the dates of an episode of care were entirely contained within another episode of care, only the latest end date was used as a service contact. Descriptive statistics provide an overview of the cohort, including age at death, underlying cause of death, drugs involved and healthcare contacts, reported as frequencies and relative frequencies, stratified by state and sex within state.

To estimate the proportion of people who died from meth/amphetamine-related causes with any service contact, the number of people with any service contact was divided by the total number of people who died from meth/amphetamine-related causes and reported as a percentage. The proportion was reported by service type and calculated across the full period before death, the year before death, and 30 days before death. Chi-square tests were performed to compare characteristics between sexes, as well as between sex and the proportion of acute healthcare contacts, with statistical significance set at *p* < 0.05.

To estimate the incidence rate of acute healthcare contacts, person-time at-risk was calculated from 1 July 2007 to the date of death and expressed in person-years. Person-years at-risk was then split into calendar years. Events were acute healthcare contacts. Overall incidence rate (and 95% CI) for acute healthcare contact was defined as the total number of acute healthcare contacts divided by the total person-years at-risk, reported per person-year. Incidence rates of acute healthcare contacts in the 12 months and 30 days prior to death were also estimated. Incidence rates of health service contacts per person-year by service type were compared between sexes using generalised linear models (Poisson distribution). Associations between calendar year (modelled as a continuous variable) and the count of health service contacts were estimated using generalised linear models (Poisson distribution), with log(person-time) included as an offset.

Acute healthcare provision differs between Australian states in terms of service structure, funding models, access pathways, and data collection systems. To avoid drawing inaccurate conclusions based on non-equivalent systems, direct comparison of acute service contacts between states was not conducted. All data management and calculations were performed using R (version 4.4.2) [24]. 

### Ethics

Ethics approval for this study was received from The Alfred Ethics Committee (127/22) and from the NSW Population & Health Services Research Ethics Committee (2022_ETH02415).

## Results

### Prevalence of deaths

A total of 2,075 meth/amphetamine-related deaths occurred between 1 July 2008 and 31 December 2021 in NSW and 2,045 in VIC (Table 1). Deaths predominantly occurred among males, comprising 76.3% of cases in NSW and 78.0% in VIC. Median age at death was similar between states (39 years in NSW and 38 years in VIC).

### Underlying cause of death

The underlying cause of death was drug-induced in 68.1% of cases in NSW and 64.9% in VIC. These deaths were largely due to accidental poisoning (59.5% NSW; 56.3% VIC), followed by drug-induced intentional self-harm deaths (5.2% NSW; 3.6% VIC). Non-drug-induced intentional self-harm contributed to 13.3% of deaths in NSW and 17.9% in VIC. Natural causes, predominantly diseases of the circulatory system, made up 11.3% of deaths in NSW and 11.2% in VIC. Unintentional injuries represented a small proportion (5.2% NSW; 4.0% VIC). Other causes accounted for approximately 2% in both states (2.1% NSW; 2.0% VIC). Opioids were detected in 48.5% of deaths in NSW and 56.7% in VIC. Cannabinoids (23.3% NSW; 30.6% VIC) and alcohol (23.3% NSW; 25.7% VIC) were also frequently detected (Table 1).

### Overall healthcare contact

Between 1 July 2007 and 31 December 2021, 92.0% of the cohort in NSW, and 91.6% in VIC had at least one acute healthcare contact. A higher proportion of females had any acute healthcare contacts compared to males in both NSW and VIC (95.1% vs. 91.0% NSW [*p* = 0.004]; 95.5% vs. 90.5% VIC [*p* = 0.002]). In the 12 months before death, the proportion of people with any acute healthcare contact remained high (69.2% in NSW and 68.0% in VIC). A higher proportion of females had any acute healthcare contact in the 12 months before death compared to males in both NSW (76.2% vs. 67.0% [p = < 0.001]) and VIC (78.6% vs. 65.0% [p = < 0.001]). In the 30 days before death, one-third (33.3%) of individuals in NSW, and 28.7% in VIC had at least one acute healthcare contact. The median days between the last acute healthcare contact and death were 110 (IQR = 411) in NSW and 112 (IQR = 391) in VIC.

**Table 1:** Characteristics of meth/amphetamine-related deaths in NSW and VIC between 1/7/2008 and 31/12/2021

	New South Wales (*N* = 2,075)			Victoria (*N* = 2,045)		
	Male	Female	Total	Male	Female	Total
Deaths (n, %)	1,583 (76.3%)	492 (23.7%)	2,075 (100.0%)	1,596 (78.0%)	449 (22.0%)	2,045 (100.0%)
Age at death (median, IQR)	39 (15.0)	39 (18.0)	39 (16.5)	37 (16.0)	39 (16.0)	38 (16.0)
**Underlying cause of death**						
Drug-induced (n, %)	**1**,**038 (65.6%)**	**376 (76.4%)**	**1**,**414 (68.1%)**	**1**,**001 (62.7%)**	**327 (72.8%)**	**1**,**328 (64.9%)**
Accidental poisoning	**923 (58.3%)**	**311 (63.2%)**	**1**,**234 (59.5%)**	**875 (54.8%)**	**277 (61.7%)**	**1**,**152 (56.3%)**
Intentional poisoning	**66** **(4.2%)**	**42** **(8.5%)**	**108** **(5.2%)**	51 (3.2%)	22 (4.9%)	73 (3.6%)
Other	49 (3.1%)	23 (4.7%)	72 (3.5%)	75 (4.7%)	28 (6.2%)	103 (5.0%)
Intentional self-harm (non-drug induced) (n, %)	**232 (14.7%)**	**43** **(8.7%)**	**275 (13.3%)**	**313 (19.6%)**	**53** **(11.8%)**	**366 (17.9%)**
Hanging	**176 (11.1%)**	**34** **(6.9%)**	**210 (10.1%)**	**231 (14.5%)**	**42** **(9.4%)**	**273 (13.3%)**
Other	56 (3.5%)	9 (1.8%)	65 (3.1%)	**82** **(5.1%)**	**11** **(2.4%)**	**93** **(4.5%)**
Natural causes (n, %)	182 (11.5%)	53 (10.8%)	235 (11.3%)	180 (11.3%)	49 (10.9%)	229 (11.2%)
Circulatory system diseases	**119** **(7.5%)**	**23** **(4.7%)**	**142** **(6.8%)**	118 (7.4%)	25 (5.6%)	143 (7.0%)
Other	63 (4.0%)	30 (6.1%)	93 (4.5%)	62 (3.9%)	24 (5.3%)	86 (4.2%)
Unintentional injury (n, %)	**95** **(6.0%)**	**12** **(2.4%)**	**107** **(5.2%)**	**72** **(4.5%)**	**9** **(2.0%)**	**81** **(4.0%)**
Other (n, %)	36 (2.3%)	8 (1.6%)	44 (2.1%)	30 (1.9%)	11 (2.4%)	41 (2.0%)
**Drugs present in death** ^**1**^						
Opioid	**740 (46.7%)**	**266 (54.1%)**	**1**,**006 (48.5%)**	**878 (55.0%)**	**281 (62.6%)**	**1**,**159 (56.7%)**
Cannabinoids	359 (22.7%)	125 (25.4%)	484 (23.3%)	506 (31.7%)	119 (26.5%)	625 (30.6%)
Alcohol	375 (23.7%)	108 (22.0%)	483 (23.3%)	420 (26.3%)	105 (23.4%)	525 (25.7%)
Antidepressants	**129** **(8.1%)**	**82** **(16.7%)**	**211 (10.2%)**	230 (14.4%)	112 (24.9%)	342 (16.7%)
Cocaine	**132** **(8.3%)**	**23** **(4.7%)**	**155** **(7.5%)**	**112** **(7.0%)**	**11** **(2.4%)**	**123** **(6.0%)**
**Any acute healthcare contact**						
Entire period before death	**1**,**440 (91.0%)**	**468 (95.1%)**	**1**,**908 (92.0%)**	**1**,**444 (90.5%)**	**429 (95.5%)**	**1**,**873 (91.6%)**
12 months before death	**1**,**060 (67.0%)**	**375 (76.2%)**	**1**,**435 (69.2%)**	**1**,**037 (65.0%)**	**353 (78.6%)**	**1**,**390 (68.0%)**
1 month before death	515 (32.5%)	176 (35.8%)	691 (33.3%)	**433 (27.1%)**	**153 (34.1%)**	**586 (28.7%)**

Note: bold denotes a significant difference between male and females (p = < 0.05); ^1^ Multiple drugs could be involved in each death

### Prevalence of healthcare contact by service type

Across the entire period prior to death, there were 13,691 ambulance attendances, 29,306 ED presentations, and 15,391 hospitalisations in NSW, and 12,133, 24,707, and 16,458, respectively in VIC (Table 2). A large majority of decedents had at least one ED presentation (89.4% NSW; 87.5% VIC), hospital admission (79.5% NSW; 80.6% VIC), or ambulance attendance (75.4% NSW; 73.2% VIC); significantly higher proportions of females than males recorded an ambulance attendance. In the 12 months before death nearly half of both jurisdictional cohorts had an ambulance attendance (47.0% NSW; 47.3% VIC), or a hospitalisation (43% of decedents in both states), while more than two-thirds (63.5% NSW; 60.3% VIC) had an ED presentation. In the 30 days before death, 18.1% of NSW cases and 19.0% in VIC had an ambulance attendance, 27.2% in NSW and 22.8% in VIC presented to the ED, and 11% were admitted to hospital (11.4% NSW; 11.4% VIC).

### Incidence rates of healthcare contact

Across both NSW and VIC, incidence rates of acute health service contacts per person-year among people who died from meth/amphetamine-related causes increased with proximity to when death occurred. ED presentations showed the largest increases, rising from 1.44 contacts per person-year (95% CI: 1.43–1.46) across the entire period, to 2.66 (95% CI: 2.59–2.73) in the year before death in NSW, and from 1.17 (95% CI: 1.16–1.19) to 2.01 (95% CI: 1.88–2.14) in VIC (Table 2). In the 30 days prior to death, ED presentation rates peaked at 5.11 contacts per person-year (95% CI: 4.78–5.45) in NSW and 4.52 (95% CI: 4.20–4.84) in VIC. Similar patterns were observed for ambulance attendances and hospitalisations. Incidence rates of acute health service contacts per person-year were higher among females compared to males for each health service across the entire period, year, and month before death in VIC. In NSW, there were no statistically significant sex differences in emergency department or hospitalisation rates in the year or month before death; in all other categories, females had higher contact rates than males.

**Table 2:** Acute healthcare contact prior to death in New South Wales and Victoria between 1/7/2007 and 31/12/2021

	New South Wales (*N* = 2,075)									Victoria (*N* = 2,045)								
	Ambulance			Emergency department			Hospital inpatients			Ambulance			Emergency department			Hospital inpatients		
	Male	Female	Total	Male	Female	Total	Male	Female	Total	Male	Female	Total	Male	Female	Total	Male	Female	Total
Total contacts (n, %)	**9**,**079 (66.3%)**	**4**,**612 (33.7%)**	**13**,**691 (100%)**	**21**,**467 (73.3%)**	**7**,**839 (26.7%)**	**29**,**306 (100%)**	**10**,**491 (68.9%)**	**4**,**726 (31.1%)**	**15**,**217 (100%)**	**7**,**909** **(65.2%)**	**4**,**224 (34.8%)**	**12**,**133 (100%)**	**16**,**825 (68.1%)**	**7**,**882 (31.9%)**	**24**,**707 (100%)**	**9**,**992 (62.0%)**	**6**,**124 (38.1%)**	**16**,**116 (100%)**
Prevalence (n, %)	**1**,**144 (72.3%)**	**420 (85.4%)**	**1**,**564 (75.4%)**	**1**,**393 (88.0%)**	**463 (94.1%)**	**1**,**856 (89.4%)**	**1**,**216 (76.8%)**	**434 (88.2%)**	**1**,**650 (79.5%)**	**1**,**161 (70.8%)**	**383 (81.8%)**	**1**,**544 (73.2%)**	**1**,**379 (86.4%)**	**410 (91.3%)**	**1**,**789 (87.5%)**	**1**,**251 (78.4%)**	**397 (88.4%)**	**1**,**648 (80.6%)**
Prevalence in year prior to death (n, %)	**694 (43.8%)**	**281 (57.1%)**	**975 (47.0%)**	**973 (61.5%)**	**344 (69.9%)**	**1**,**317 (63.5%)**	**633 (40.0%)**	**256 (52.0%)**	**889 (42.8%)**	**730 (44.5%)**	**268 (57.3%)**	**998 (47.3%)**	**919 (57.6%)**	**314 (69.9%)**	**1**,**233 (60.3%)**	**637 (39.9%)**	**241 (53.7%)**	**878 (42.9%)**
Prevalence in month prior to death (n, %)	**267 (16.9%)**	**108 (22.0%)**	**375 (18.1%)**	**412 (26.0%)**	**153 (31.1%)**	**565 (27.2%)**	173 (10.9%)	63 (12.8%)	236 (11.4%)	**292 (17.8%)**	**108 (23.1%)**	**400 (19.0%)**	**343 (21.5%)**	**124 (27.6%)**	**467 (22.8%)**	**163 (10.2%)**	**71 (15.8%)**	**234 (11.4%)**
Incidence rate (contacts per person year, 95% CI)	**0.58 (0.57–0.60)**	**0.97 (0.94–0.99)**	**0.67 (0.66–0.69)**	**1.38 (1.36–1.40)**	**1.64 (1.61–1.68)**	**1.44 (1.43–1.46)**	**0.67 (0.66–0.69)**	**0.99 (0.96–1.02)**	**0.75 (0.74–0.76)**	**0.48 (0.47–0.49)**	**0.91 (0.89–0.94)**	**0.58 (0.56–0.59)**	**1.02 (1.01–1.04)**	**1.71 (1.67–1.75)**	**1.17 (1.16–1.19)**	**0.61 (0.59–0.62)**	**1.33 (1.29–1.36)**	**0.76 (0.75–0.78)**
Incidence rate in year prior to death (contacts per person year, 95% CI)	**1.24 (1.18–1.29)**	**1.77 (1.65–1.89)**	**1.37 (1.32–1.42)**	2.67 (2.59–2.75)	2.65 (2.50–2.79)	2.66 (2.59–2.73)	**1.25 (1.20–1.31)**	**1.47 (1.36–1.57)**	**1.30 (1.25–1.35)**	**1.25 (1.20–1.30)**	**1.93 (1.81–2.06)**	**1.40 (1.35–1.45)**	**2.12 (2.05–2.19)**	**3.18 (3.02–3.35)**	**2.01 (1.88–2.14)**	**1.10 (1.05–1.15)**	**2.14 (2.01–2.28)**	**1.33 (1.28–1.38)**
Incidence rate in month prior to death (contacts per person year, 95% CI)	**2.65 (2.38–2.93)**	**3.67 (3.08–4.25)**	**2.89 (2.64–3.15)**	5.13 (4.74–5.51)	5.07 (4.38–5.75)	5.11 (4.78–5.45)	2.17 (1.92–2.42)	2.19 (1.74–2.65)	2.17 (1.95–2.39)	**2.86 (2.58–3.14)**	**4.29 (3.64–4.93)**	**3.17 (2.91–3.44)**	**4.04 (3.70–4.38)**	**6.24 (5.44–7.03)**	**4.52 (4.20–4.83)**	**1.61 (1.40–1.82)**	**2.51 (2.02-3.00)**	**1.81 (1.61-2.00)**

Note: bold denotes a significant difference between male and females (p = < 0.05)

### Trends in incidence rates of healthcare contacts

**Fig. 1:**
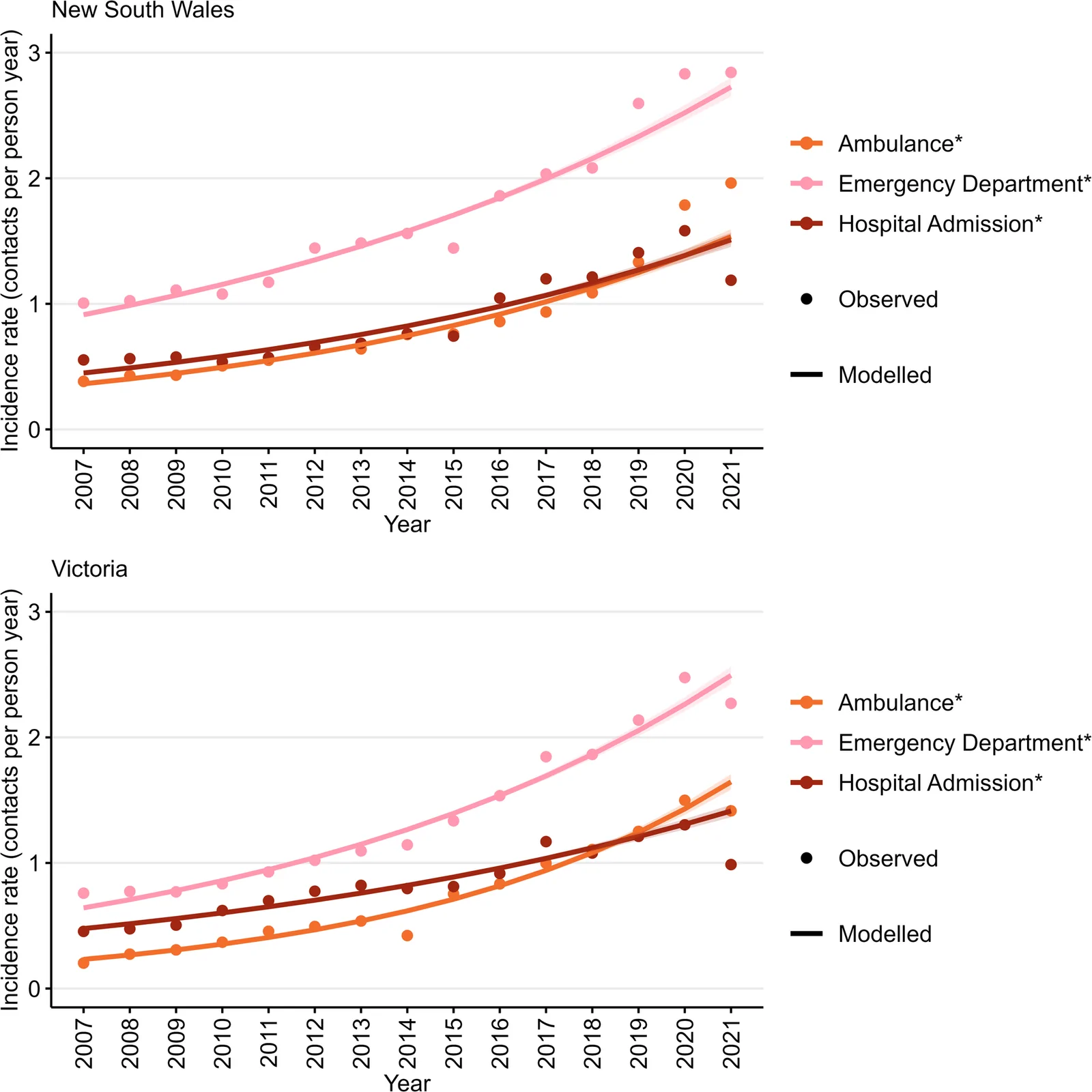
Incidence rate of acute healthcare contacts per person-year before meth/amphetamine-related death, NSW and VIC. Note: An * denotes a significant increase in incidence rates over time (p = < 0.05)

The annual incidence rate of acute healthcare contacts among people who died from meth/amphetamine-related causes increased over time (see Fig. 1 and Supplementary Tables 4 and 5). In NSW, the incidence rate of ambulance attendances (incidence rate ratio (IRR) = 1.11, 95% CI: 1.10–1.11, *p* < 0.005), ED presentations (IRR = 1.08, 95% CI: 1.08–1.08, *p* < 0.005), and hospitalisations (IRR = 1.09, 95% CI: 1.09–1.10, *p* < 0.005) all increased significantly over time. The incidence rate of ambulance attendances (IRR = 1.15, 95% CI: 1.14–1.16, *p* < 0.005), ED presentations (IRR = 1.10, 95% CI: 1.10–1.11, *p* < 0.005), and hospitalisations (IRR = 1.08, 95% CI: 1.08–1.09, *p* < 0.005) also all increased significantly over time in VIC. The incidence rates in the 12 months prior to death increased in a similar pattern to the annual rates in both NSW and VIC (see Supplementary Fig. 2). Annual incidence rates of acute healthcare contacts were consistently higher among females compared to males across all service types and states (see Supplementary Figs. 3–8).

## Discussion

This study found that over 90% of people who died from meth/amphetamine-related causes in NSW and Victoria had had at least one ambulance, ED, or hospital admission contact prior to death. Two-thirds had contact in the year prior to death, and a third had contact in the month prior to death. Analysis of these contacts revealed that ED presentations are the most prevalent entry point to acute care. High prevalence of acute healthcare contacts prior to meth/amphetamine-related death is consistent with international evidence. Studies from Canada, the United States, and Taiwan have similarly found that a high proportion of people who die from stimulant-related causes engaged with hospitals, emergency departments, and ambulance services at least once before death. Although the current study includes all cause meth/amphetamine related deaths (including suicide), the prevalence of prior year use of any acute health service was lower than reported in a Taiwanese study of people with a meth/amphetamine use disorder who died by suicide, where 87% used a health service in the three months before suicide [17]. This could point to differences in healthcare utilisation depending on the underlying cause of death, as the prevalence of people who die by suicide accessing health services is shown to be high [25]. These convergent findings show that people who die from meth/amphetamine-related causes are often not “hidden” from healthcare systems, as most decedents have multiple contacts with acute healthcare services.

Incidence rates of ambulance attendances, ED presentations, and hospitalisations increased noticeably in the year and month before death. This pattern of escalation is consistent with the broader population but is far more pronounced, particularly for emergency department presentations [26, 27]. Among people who died from all causes in Australia between 2010 and 2016, the average annual number of ED presentations per person was 1.4, and 2.6 hospitalisations [28]. These findings contrast with the pattern observed prior to meth/amphetamine-related deaths, where EDs were accessed more frequently than hospitalisations. This may reflect the acute and episodic nature of meth/amphetamine-related harms that prompt ED presentations that resolve without requiring admission, as well as the younger age profile of those who die from meth/amphetamine-related causes [9, 29]. Among other drug-related deaths, the frequency of health service use appears relatively high. A Welsh study of people who died from opioid overdose between 2012 and 2018 found that in the three years prior to death, the median ED presentation per person was double the median hospitalisations per person (4 vs. 2) [30]. Similarly, a North American study reported a mean of 2.3 emergency medical service attendances per person among individuals who died from opioid overdose [31]. Our findings that females had higher incidence rates of service use across in most service settings in both states align with other Australian research, which found that females who use meth/amphetamine had higher health service use costs than males [32]. Further investigation into the underlying drivers of these sex differences is warranted.

The increasing rates of acute healthcare service contact over time and in the year prior to death among people who died from meth/amphetamine-related causes in NSW and VIC likely reflects a convergence of market changes (notably, increased purity), evolving use patterns and increasingly complex health profiles of older Australians who are at greatest risk of meth/amphetamine-related death [9]. Since 2010 there have been significant changes in patterns of meth/amphetamine use, with an increase in regular use of high-purity crystal meth/amphetamine, which is known to be associated with greater harms including a higher risk of dependence [18, 33–35]. Increased availability of meth/amphetamine at lower prices may also have contributed to rising dependence and increased acute health service use before death [35, 36]. These factors may help explain the upward trend in rates of acute service use observed in this older cohort of decedents, and mirror broader trends in health service engagement among people who use meth/amphetamine in Australia over time. Australian research has noted increases in meth/amphetamine-related ambulance attendances in Victoria in the 2010s, where attendances increased from 768 in 2011–12 to 2,514 in 2016–17, reflecting the growing burden on emergency medical services [37]. Similarly, meth/amphetamine-related ED presentations in NSW increased from 470 in 2009–10 to 9,098 in 2019–20, and meth/amphetamine-related emergency presentations increased from 651 in 2010–11 to 9,261 in 2019–20 [38, 39]. These findings highlight the increasing pressure meth/amphetamine use and related harms have placed on acute healthcare services.

A small proportion of decedents (between 8 and 9%) had no recorded contact with ambulance, emergency, or hospital services prior to death. This same trend is seen internationally, with a recent latent class analysis of care contacts in the year before death among people who died from opioid overdose in Sweden identifying a small proportion of the cohort with limited interaction with any services [40]. The lack of interaction with services may reflect barriers to accessing care, such as stigma, mistrust, or absence of culturally appropriate services [41, 42]. A Canadian qualitative study involving people with lived and living experience of meth/amphetamine use who had previously been hospitalised found that frequent negative interactions with hospital staff contributed to distrust of the healthcare system and avoidance of attending care [43]. Similarly, an Australian survey of people who use meth/amphetamine found that fear of seeking help and fear of being judged by others were the main barriers to accessing support [41]. Another explanation for the lack of prior contact is the occurrence of sudden cardiac events among people who use meth/amphetamine who otherwise may not have experienced harms related to their use. Meth/amphetamines can quickly raise heart rate and blood pressure, and sometimes cause fatal cardiac arrhythmias or stroke, even in individuals with no previous warning signs [44, 45]. 

### Policy implications

A key insight from our study is that many people who have died from meth/amphetamine-related causes frequently access the acute healthcare system in Australia, particularly in the month prior to death. Research in Australia has highlighted that better provision of non-acute health services (such as community mental health, primary care, harm reduction and drug treatment services) for people who use meth/amphetamine can reduce the burden on acute services [10]. This suggests that if low-threshold services were available and acceptable to this population, some crises leading to acute care could be avoided [46]. Investment in harm reduction and treatment services may turn out to be cost saving in the long term by preventing more expensive acute health contacts and mortality.

Given that a large proportion of people who died from meth/amphetamine-related causes had contact in acute care settings in the year prior to death, these services represent crucial intervention points for the provision of further care that may reduce deaths in the future. The transition from acute to community care is a vulnerable period for people with complex or frequent health needs, due to the risk of fragmented care, limited follow-up, and challenges navigating multiple services [47]. Strengthening continuity-of-care interventions that begin in hospitals and connect patients to post-discharge services is critical. For example, the evaluation of the “BEAT Meth” program in the US, which piloted an ED intervention for meth/amphetamine-related psychosis, found that patients who received follow-up support were nearly three times more likely (32% vs. 11%) to attend a drug treatment appointment post-discharge [48]. As people who use meth/amphetamine frequently attend acute health services, treating their health issues in an integrated fashion, and then actively connecting them to ongoing care, healthcare systems may mitigate the risk of subsequent fatal outcomes.

The study’s use of linked administrative health databases highlights the value of integrating information across systems. To systematically respond to rising meth/amphetamine-related harms, improved linkages between acute health services, community mental health, general practitioners, and alcohol and other drug treatment services are needed. Integrated data systems have been implemented internationally to detect drug-related overdoses and show promise in enhancing the identification and prioritisation of individuals in need of care [49]. By sharing data across health systems, people with complex or frequent health needs can be flagged for follow-up regardless of where they engage with the system. Integrated data systems can also incorporate advanced machine‑learning algorithms to provide clinical decision support tools [50, 51]. In the US, machine learning tools are currently being trialled in primary care settings to identify patients at elevated risk of opioid overdose within three months of being prescribed an opioid, prompting consideration of naloxone co-prescribing and medication adjustment [52]. Advanced integrated data systems and the application of machine learning techniques have yet to be explored for meth/amphetamine-related harm prevention in Australia.

### Limitations

This study is subject to several limitations inherent to the use of administrative data. The cause of death data used in this study are compiled from the national coronial system and coded based on the information available at the time. Differences in the type and amount of information available can lead to coding variation between jurisdictions, and coding practices may also change over time. Probabilistic linkage may result in false-positive or false-negative matches, particularly when identifying information is missing or inconsistent. Linkage was undertaken by specialist data linkage units the CHeReL in NSW and the CVDL in Victoria. Both linkage units reported that linkage parameters were set to minimise missed true matches where full identifiers were available, and estimated the false positive rate to be less than 1% across both cohorts. Missed links may have resulted in under-ascertainment of acute healthcare contact, particularly for people with incomplete identifiers, however this is likely to be minimal. ED databases in both states do not include presentations to private hospitals, potentially underestimating acute health service use in both states. The decline in incidence rate for ambulance attendances in VIC in 2014 is likely due to a period of protected industrial action, where paramedics did not complete details on electronic case records [53]. Patterns of acute healthcare contact observed in 2020 and 2021 may have been influenced by the COVID-19 pandemic, which affected healthcare-seeking behaviour, ambulance demand, ED attendances, admission practices, and discharge pathways [54, 55]. Analysis was restricted to service contacts within the state where the person died, meaning that for individuals who moved between states in Australia or received care in another jurisdiction, their full acute health service use history may not be captured. Although all individuals had one year of follow-up, those who died in the early years of the study may have had fewer opportunities to appear in administrative records, potentially leading to an underestimation of overall prevalence figures. Without a comparison group of non-decedents or people who use meth/amphetamine, it is not possible to determine whether observed healthcare use patterns are specific to those who died.

## Conclusion

This study provides a comprehensive analysis of acute healthcare utilisation prior to meth/amphetamine-related deaths in Australia’s two most populous states. It reveals that the majority of people who died from meth/amphetamine-related causes had at least one contact with acute healthcare services, especially with EDs. The incidence rate of acute healthcare contacts before death highlights that many individuals sought help multiple times. Each interaction is a unique opportunity for support. Moving forward, the challenge is to better meet people where they are, with integrated and stigma-free healthcare provision that genuinely engages and supports people. By understanding how and when people access ambulances, EDs, and hospitals before death, health systems can respond more effectively to reduce meth/amphetamine-related deaths in Australia.

## Supplementary Information

Below is the link to the electronic supplementary material.


Supplementary Material 1


## Data Availability

The datasets analysed during the current study are not publicly available due to containing personal and sensitive information, and restrictions under ethical approvals and privacy regulations.

## Abbreviations

ED: emergency department
GHB: gamma-hydroxybutyrate
MDMA: 3,4-methylenedioxymethamphetamine
NSW: New South Wales
VIC: Victoria
ICD-10: International Classification of Diseases, Tenth Revision
CHeReL: Centre for Health Records Linkage
CVDL: The Centre for Victorian Data Linkage
IRR: Incidence rate ratio
