# Supplementary material for: Acute health service use among people who died from methamphetamine-related causes in New South Wales and Victoria
Source: BMC Health Serv Res. 2026 Jun 18;26:1205. doi: 10.1186/s12913-026-14952-x (PMC13527950; doi:10.1186/s12913-026-14952-x)
Supplement: Supplementary file 1 [file 12913_2026_14952_MOESM1_ESM.docx]

**Acute health service use among people who died from methamphetamine-related causes in New South Wales and Victoria**

**Supplementary Materials**

**Table of Contents**

[Supplementary Table 1. ICD–10 codes used to identify the cohort of people who died from meth/amphetamine related causes in New South Wales (NSW) and Victoria (VIC).	2](#_Toc229042552)

[Supplementary Table 2. ICD–10 codes used to categorise underlying cause of meth/amphetamine-related death in NSW and VIC.	2](#_Toc229042553)

[Supplementary Figure 1. Linked data flow for NSW and VIC in the overall project.	3](#_Toc229042554)

[Supplementary Table 3. Linked datasets in NSW and VIC	4](#_Toc229042555)

[Supplementary Table 4. Observed incidence rate (IR) of acute healthcare contacts per person-year before meth/amphetamine-related death NSW and VIC.	6](#_Toc229042556)

[Supplementary Figure 1. Incidence rate of acute health service contacts per person year in the year prior to meth/amphetamine-related death by year in NSW (top) and VIC (bottom).	7](#_Toc229042557)

[Supplementary Table 5. Observed incidence rate (IR) of acute healthcare contacts per person-year in the year before meth/amphetamine-related death NSW and VIC.	8](#_Toc229042558)

[Supplementary Figure 2. Incidence rates of acute Ambulance attendances per person year before meth/amphetamine-related death by year and sex in NSW.	9](#_Toc229042559)

[Supplementary Figure 3. Incidence rates of acute Ambulance attendances per person year before meth/amphetamine-related death by year and sex in VIC.	9](#_Toc229042560)

[Supplementary Figure 4. Incidence rates of emergency department presentations per person year before meth/amphetamine-related death by year and sex in NSW.	10](#_Toc229042561)

[Supplementary Figure 5. Incidence rates of emergency department presentations per person year before meth/amphetamine-related death by year and sex in VIC.	10](#_Toc229042562)

[Supplementary Figure 6. Incidence rates of hospital admissions per person year before meth/amphetamine-related death by year and sex in NSW.	11](#_Toc229042563)

[Supplementary Figure 7. Incidence rates of hospital admissions per person year before meth/amphetamine-related death by year and sex in VIC.	11](#_Toc229042564)

# Supplementary Table 1. ICD–10 codes used to identify the cohort of people who died from meth/amphetamine related causes in New South Wales (NSW) and Victoria (VIC).

| **UCOD** (ICD–10 code/s) | | **MCOD** (ICD–10 code/s) |
| --- | --- | --- |
| *Deaths where methamphetamines are the underlying cause of death* | | |
| F15 | | X41 |
| X41 | | T43.6 |
| X61 | | T43.6 |
| Y11 | | T43.6 |
| F15 | | T43.6 |
| *Deaths where methamphetamines are contributory* | | |
| Any | T436 OR F15 | |

# Supplementary Table 2. ICD–10 codes used to categorise underlying cause of meth/amphetamine-related death in NSW and VIC.

| **Circumstances of death** | | **ICD–10 code/s** |
| --- | --- | --- |
| **Drug induced** | |  |
|  | Unintentional drug toxicity | X40–X44 |
|  | Intentional self–harm | X60–X64 |
|  | Other | D52.1, D59.0, D59.2, D61.1, D64.2, E06.4, E16.0, E23.1, E24.2, E27.3, E66.1, F11.0–F11.5, F11.7–F11.9, F12.0–F12.5, F12.7–F12.9, F13.0–F13.5, F13.7–F13.9, F14.0–F14.5, F14.7–F14.9, F15.0–F15.5, F15.7–F15.9, F16.0–F16.5, F16.7–F16.9, F18.0–F18.5, F18.7–F18.9, F19.0–F19.5, F19.7–F19.9, G21.1, G24.0, G25.1, G25.4, G25.6, G44.4, G62.0, G72.0, I95.2, J70.2, J70.3, J70.4, L10.5, L27.0, L27.1, M10.2, M32.0, M80.4, M81.4, M83.5, M87.1, R78.1, R78.2, R78.3, R78.4, R78.5, X85, Y10–Y14 |
| **Intentional self–harm (non–drug induced)** | |  |
|  | Hanging | X70 |
|  | Other | X65–69, X71–X84 |
| **Unintentional injury** | | V00–V99, W00–W99, X00–X39 |
| **Natural causes** | |  |
|  | Circulatory system diseases | I00–I99 |
|  | Other | D50–99, H00–99, L00–99, M00–99, N00–99, O00–99, P00–99, Q00–99, U00–99 |
| **Other** | | X85–X99, Y00–Y09, R00–99, Y15–99, F00–99, X45–59 |

# Supplementary Figure 1. Linked data flow for NSW and VIC in the overall project.

# Supplementary Table 3. Linked datasets in NSW and VIC

| **State** | **Dataset** | **Description** |
| --- | --- | --- |
| NSW | Cause of Death Unit Record File (CODURF) | The CODURF holds details of all registered deaths in NSW. A single code for an underlying cause of death is applied. Multiple contributory causes of death codes (ICD-10) were applied to each death record where more than one cause contributed to the death. |
| NSW | Admitted Patient Data Collection (APDC) | The APDC contains records of all inpatient separations (discharges, transfers and deaths) from all public, private, psychiatric and repatriation hospitals in NSW as well as public multipurpose services, private day procedure centres and public nursing homes. |
| NSW | Emergency Department Data Collection (EDDC) | The EDDC provides information about patient presentations to the emergency departments of public hospitals in NSW. |
| NSW | Ambulance |  |
|  | Computer Aided Dispatch (CAD) | The CAD is a centralised state-wide emergency and patient transport call-taking and dispatch system for the logging and allocation of resources to Triple Zero calls. |
|  | Patient Health Care Record (PHCR) | PHCR includes paper data collected during the patient care episode, recorded by paramedics. This includes information about the incident, patient information, treatment details, and outcomes. |
|  | Electronic Medical Record (eMR) | The eMR includes electronic data collected during the patient care episode, recorded by paramedics. This includes information about the incident, patient information, treatment details, and outcomes. |
| VIC | Victorian Death Register COD (VDICOD) | The VDICOD holds details of all registered deaths in VIC. A single code for an underlying cause of death is applied. Multiple contributory causes of death codes (ICD-10) were applied to each death record where more than one cause contributed to the death. The dataset includes both coronial and registry data. |
| VIC | Victorian Admitted Episodes Dataset (VAED) | VAED provides a comprehensive dataset of the causes, effects, nature of illness and immediate outcome following treatment, and the use of hospital health services in Victoria. |
| VIC | Victorian Emergency Minimum Dataset (VEMD) | The VEMD comprises demographic, administrative and clinical data detailing presentations at Victorian public hospitals with designated emergency departments. The VEMD does not capture emergency department presentations at private hospitals. |
| VIC | Victorian Ambulance Data (VACIS) | VACIS contains data on emergency and urgent episodes of care provided by Ambulance Victoria, including whether people were transported to hospital, left at the scene or died at the scene. |

# Supplementary Table 4. Observed incidence rate (IR) of acute healthcare contacts per person-year before meth/amphetamine-related death NSW and VIC.

| **Year** | **NSW**  (IR [95% CI]) | | | **VIC**  (IR [95% CI]) | | |
| --- | --- | --- | --- | --- | --- | --- |
|  | **Ambulance** | **ED** | **Hospital** | **Ambulance** | **ED** | **Hospital** |
| 2007 | 0.4 (0.3–0.4) | 1.0 (0.9–1.1) | 0.6 (0.5–0.6) | 0.2 (0.2–0.2) | 0.8 (0.7–0.8) | 0.5 (0.4–0.5) |
| 2008 | 0.4 (0.4–0.5) | 1.0 (1.0–1.1) | 0.6 (0.5–0.6) | 0.3 (0.3–0.3) | 0.8 (0.7–0.8) | 0.5 (0.4–0.5) |
| 2009 | 0.4 (0.4–0.5) | 1.1 (1.1–1.2) | 0.6 (0.5–0.6) | 0.3 (0.3–0.3) | 0.8 (0.7–0.8) | 0.5 (0.5–0.5) |
| 2010 | 0.5 (0.5–0.5) | 1.1 (1.0–1.1) | 0.5 (0.5–0.6) | 0.4 (0.3–0.4) | 0.8 (0.8–0.9) | 0.6 (0.6–0.7) |
| 2011 | 0.6 (0.5–0.6) | 1.2 (1.1–1.2) | 0.6 (0.5–0.6) | 0.5 (0.4–0.5) | 0.9 (0.9–1.0) | 0.7 (0.7–0.7) |
| 2012 | 0.6 (0.6–0.7) | 1.4 (1.4–1.5) | 0.7 (0.6–0.7) | 0.5 (0.5–0.5) | 1.0 (1.0–1.1) | 0.8 (0.7–0.8) |
| 2013 | 0.6 (0.6–0.7) | 1.5 (1.4–1.5) | 0.7 (0.6–0.7) | 0.5 (0.5–0.6) | 1.1 (1.0–1.1) | 0.8 (0.8–0.9) |
| 2014 | 0.8 (0.7–0.8) | 1.6 (1.5–1.6) | 0.8 (0.7–0.8) | 0.4 (0.4–0.5) | 1.1 (1.1–1.2) | 0.8 (0.8–0.8) |
| 2015 | 0.8 (0.7–0.8) | 1.4 (1.4–1.5) | 0.7 (0.7–0.8) | 0.8 (0.7–0.8) | 1.3 (1.3–1.4) | 0.8 (0.8–0.9) |
| 2016 | 0.9 (0.8–0.9) | 1.9 (1.8–1.9) | 1.0 (1.0–1.1) | 0.8 (0.8–0.9) | 1.5 (1.5–1.6) | 0.9 (0.9–1.0) |
| 2017 | 0.9 (0.9–1.0) | 2.0 (2.0–2.1) | 1.2 (1.1–1.3) | 1.0 (0.9–1.1) | 1.8 (1.8–1.9) | 1.2 (1.1–1.2) |
| 2018 | 1.1 (1.0–1.2) | 2.1 (2.0–2.2) | 1.2 (1.1–1.3) | 1.1 (1.0–1.2) | 1.9 (1.8–2.0) | 1.1 (1.0–1.1) |
| 2019 | 1.3 (1.2–1.4) | 2.6 (2.5–2.7) | 1.4 (1.3–1.5) | 1.3 (1.2–1.3) | 2.1 (2.0–2.2) | 1.2 (1.1–1.3) |
| 2020 | 1.8 (1.6–1.9) | 2.8 (2.6–3.0) | 1.6 (1.4–1.7) | 1.5 (1.4–1.6) | 2.5 (2.3–2.6) | 1.3 (1.2–1.4) |
| 2021 | 2.0 (1.6–2.3) | 2.8 (2.5–3.2) | 1.2 (0.9–1.4) | 1.4 (1.2–1.6) | 2.3 (2.0–2.5) | 1.0 (0.8–1.1) |

# Supplementary Figure 1. Incidence rate of acute health service contacts per person year in the year prior to meth/amphetamine-related death by year in NSW (top) and VIC (bottom).

**Note:** Across the entire study period, 3,848 ED presentations and 3,119 hospitalisations included only ICD-10-CM Z codes or ICD-9-CM V codes for factors influencing health status and contact with health services without an accompanying diagnosis code. In the year prior to death, the increase in emergency department presentations in NSW in 2012 is attributable to a small group of individuals in rural areas who regularly received methadone through an emergency department. Similarly, the rise in hospital admissions in VIC in 2013 reflects a small number of people who were frequently admitted for dialysis.

# Supplementary Table 5. Observed incidence rate (IR) of acute healthcare contacts per person-year in the year before meth/amphetamine-related death NSW and VIC.

| **Year** | **NSW**  (IR [95% CI]) | | | **VIC**  (IR [95% CI]) | | |
| --- | --- | --- | --- | --- | --- | --- |
|  | **Ambulance** | **ED** | **Hospital** | **Ambulance** | **ED** | **Hospital** |
| 2009 | 0.8 (0.4–1.1) | 1.5 (1.0–2.0) | 1.6 (1.1–2.1) | 0.9 (0.5–1.2) | 2.0 (1.5–2.6) | 0.9 (0.6–1.3) |
| 2010 | 1.2 (0.9–1.5) | 2.0 (1.6–2.4) | 1.9 (1.5–2.3) | 0.9 (0.5–1.2) | 1.0 (0.7–1.4) | 1.0 (0.7–1.4) |
| 2011 | 1.0 (0.7–1.2) | 1.7 (1.3–2.0) | 0.7 (0.4–0.9) | 1.1 (0.8–1.5) | 2.3 (1.8–2.7) | 0.7 (0.4–1.0) |
| 2012 | 1.4 (1.2–1.7) | 5.7 (5.2–6.2) | 1.1 (0.9–1.3) | 0.7 (0.5–0.9) | 1.6 (1.2–2.0) | 0.8 (0.6–1.1) |
| 2013 | 0.8 (0.6–1.0) | 1.6 (1.3–1.8) | 0.7 (0.5–0.8) | 1.9 (1.6–2.2) | 2.0 (1.6–2.3) | 3.3 (2.9–3.8) |
| 2014 | 0.9 (0.7–1.1) | 1.9 (1.6–2.1) | 0.9 (0.8–1.1) | 1.0 (0.8–1.3) | 1.8 (1.5–2.1) | 1.0 (0.8–1.2) |
| 2015 | 1.4 (1.2–1.5) | 2.4 (2.1–2.6) | 1.2 (1.0–1.3) | 1.1 (0.9–1.2) | 1.9 (1.7–2.1) | 1.2 (1.0–1.3) |
| 2016 | 1.2 (1.1–1.4) | 2.0 (1.8–2.2) | 1.0 (0.9–1.1) | 1.0 (0.8–1.1) | 1.5 (1.4–1.7) | 0.9 (0.8–1.0) |
| 2017 | 1.0 (0.9–1.1) | 2.9 (2.7–3.1) | 1.3 (1.2–1.4) | 1.5 (1.4–1.7) | 2.7 (2.5–2.9) | 2.0 (1.8–2.1) |
| 2018 | 1.4 (1.2–1.5) | 2.7 (2.5–2.9) | 1.6 (1.5–1.8) | 1.8 (1.6–1.9) | 2.9 (2.7–3.1) | 1.7 (1.5–1.8) |
| 2019 | 1.5 (1.4–1.7) | 2.9 (2.7–3.1) | 1.3 (1.1–1.4) | 1.8 (1.7–2.0) | 2.7 (2.5–2.9) | 1.3 (1.2–1.5) |
| 2020 | 1.9 (1.8–2.1) | 3.2 (3.0–3.4) | 2.0 (1.8–2.2) | 1.6 (1.5–1.8) | 3.0 (2.8–3.2) | 1.4 (1.3–1.6) |
| 2021 | 1.7 (1.5–1.9) | 2.6 (2.3–2.8) | 1.2 (1.0–1.3) | 1.6 (1.4–1.7) | 2.3 (2.1–2.5) | 1.0 (0.9–1.1) |

# Supplementary Figure 2. Incidence rates of acute Ambulance attendances per person year before meth/amphetamine-related death by year and sex in NSW.

# Supplementary Figure 3. Incidence rates of acute Ambulance attendances per person year before meth/amphetamine-related death by year and sex in VIC.

# Supplementary Figure 4. Incidence rates of emergency department presentations per person year before meth/amphetamine-related death by year and sex in NSW.

# Supplementary Figure 5. Incidence rates of emergency department presentations per person year before meth/amphetamine-related death by year and sex in VIC.

# Supplementary Figure 6. Incidence rates of hospital admissions per person year before meth/amphetamine-related death by year and sex in NSW.

# Supplementary Figure 7. Incidence rates of hospital admissions per person year before meth/amphetamine-related death by year and sex in VIC.
